# Association between opioid dependence and scale free fractal brain activity: an EEG study

**DOI:** 10.3390/fractalfract7090659

**Published:** 2023-08-31

**Authors:** Parikshat Sirpal, William A. Sikora, Desiree R. Azizoddin, Hazem H. Refai, Yuan Yang

**Affiliations:** 1 University of Oklahoma, School of Electrical and Computer Engineering, Gallogly College of Engineering, Norman, Oklahoma, United States; 2 University of Oklahoma, Stephenson School of Biomedical Engineering, Tulsa, Oklahoma, United States; 3 TSET Health Promotion Research Center, Stephenson Cancer Center, University of Oklahoma Health Sciences Center, Oklahoma City, OK 73104, USA; 4 University of Illinois Urbana-Champaign, Department of Bioengineering, Urbana, Illinois, United States; 5 Carle Foundation Hospital, Clinical Imaging Research Center, Stephenson Family Clinical Research Institute, Urbana, Illinois, United States; 6 University of Illinois Urbana-Champaign, Beckman Institute for Advanced Science and Technology, Urbana, Illinois, United States; 7 Northwestern University, Department of Physical Therapy and Human Movement Sciences, Chicago, Illinois, United States

**Keywords:** Dynamical systems, nonlinear analysis, multifractal analysis, Hurst dimension, EEG, EEG biomarker, brain networks, opioid use, pain processing

## Abstract

Self-similarities at different time scales embedded within a self-organizing neural manifold are well recognized. In this study, we hypothesize that the Hurst fractal dimension (HFD) of the scalp electroencephalographic (EEG) signal reveals statistical differences between chronic pain and opioid use. We test this hypothesis by using EEG resting state signals acquired from a total of 23 human subjects: 14 with chronic pain, 9 with chronic pain taking opioid medications, 5 with chronic pain and not taking opioid medications, and 9 healthy controls. Using the multifractal analysis algorithm, the HFD for full spectrum EEG and EEG frequency band time series was computed for all groups. Our results indicate the HFD varies spatially and temporally across all groups and is of lower magnitude in patients not taking opioids as compared to those taking opioids and healthy controls. A global decrease in HFD was observed with changes in gamma and beta power in the chronic pain group compared to controls and when paired to subject handedness and sex. Our results show the loss of complexity representative of brain wide dysfunction and reduced neural processing can be used as an EEG biomarker for chronic pain and subsequent opioid use.

## 1. Introduction

Recordings from the scalp electroencephalogram (EEG) remain a fundamental tool to understand and uncover physiological and pathological brain processes and dynamics. Advances in mathematical modeling and analysis applied to such signals can shed new light on a wide array of neurological disorders including but not limited to epilepsy, neuropsychiatric, sleep, and neurovascular (i.e., stroke) disorders to name a few [1]–[5]. Recently, there has been a focus on the on-going opioid crisis, characterized by the widespread misuse, abuse, and addiction to opioid drugs, resulting in a significant increase in opioid-related overdoses, and ultimately death [6], [7]. As the crisis worsens, related overdoses and morbidity surge globally, causing significant social and economic consequences including strained healthcare systems.

The analysis and characterization of scalp EEG signals in patients who are actively taking opioid medications to manage chronic pain provides new perspectives on substance abuse effects and addiction pathways in the brain. In fact, drug addiction and downstream brain effects are suggested to be aligned with reward related behavior and emerge from the dynamic interplay between large neural networks as opposed to a single brain structure. Investigations into the functional organization of reward and addiction brain areas is understood in the context of extended and functionally connected neural systems and the key components they form [8]. Fundamental research examining network interactions between cortical neuronal assemblies and the effects of substance abuse and addiction can be aided by the development of a noninvasive, cost-effective, and reliable biomarker of opioid addiction and abuse.

Advances in mathematical approaches allow us to better understand the inherent chaotic nature of the brain, for instance algorithms such as wavelet Jensen–Shannon divergence, the Neyman-Pearson criteria with respect to approximate entropy, multifractal detrended fluctuation analysis (MFDFA), Hausdorff fractal dimension, in EEG signal analysis [1], [9]–[13]. Here, we utilize the generalized Hurst fractal dimension (HFD) exponent to characterize multifractal patterns in resting state EEG signals of patients with chronic pain and opioid dependence, and healthy control patients. A scale free analysis of the EEG signal using the Hurst fractal dimension exponent and computation of the ${q\;}^{th}$ order moments help to determine its scaling properties. The generalized Hurst exponent (GHE) quantifies long-term memory and autocorrelation in the EEG signal at varying scales [14]–[16]. A particular advantage of the GHE approach with respect to EEG signals is that at each q scale, an estimate of the Hurst exponent value is made, allowing for computational efficiency combined with sensitivity to EEG signal dependency.

Previously, GHE estimates have been found to be consistent with other scale free methods such as multifractal detrended fluctuation analysis (MFDA) [14]–[17]. As an added advantage, results derived from GHE offer a narrower confidence interval and are robust to heavier tails [18]and do not underestimate expected values. In addition, utilizing the GHE methodology to estimate fractal measures has been effective in characterizing neurological disorders such as glioma, neuropsychiatric disorders, and epilepsy [15], [19]. Since long range temporal correlations exist typically in EEG signals, the GHE method helps describe the irregular, nonlinear dynamics present within such signals and allows for an efficient estimation of scale free fractals with limited computational burden and high efficiency. We anticipate that the GHE method will prove fruitful in computing multi-scale fractals to characterize scalp EEG signals in chronic pain and opioid use patients. Here, we implement the GHE technique along with robust statistical validation using parametric and non-parametric tests as a framework to understand the phenomenology and the information content through time in EEG signals from chronic pain opioid dependent patients and healthy control EEG signals. We further correlate our results with subject parameters to develop a fractal-based fingerprint of opioid use using scalp EEG signals.

## 2. Materials and Methods

### 2.1 Chronic Pain Patients

Fourteen patients with chronic pain (8 M, 6 F median age of 61 ± 2 years) were enrolled in this study. Within the chronic pain group, nine patients were taking opioid medications (4 M, 5 F median age of 57 ± 1.4 years), and five patients (3 M, 2 F median age of 66 ± 1.31 years) with chronic pain were not taking opioid medications. The study inclusion criteria were as follows: 1) No history of a chronic neurological disorder that limits the use of EEG equipment, including but not limited to epilepsy and chronic seizures, 2) No active history of mental disorders, 3) Not actively using a pacemaker or other such cardiac pacing device, 4) No metal implants in the head, 5) No known adverse reaction to non-invasive brain recording, 6) Absence of concurrent and comorbid medical problems (e.g., cardiorespiratory impairment, organ failure), 7) Absence of significant sensory deficits, 8) No prior history of substance addiction, 9) No previous history of brain surgery including craniotomy, 10) Study participants were not receiving any pharmacological treatment for other comorbidities (i.e., cardiovascular disease, kidney disease) at the time of the study. Participant handedness was tested using the Edinburgh Handedness Inventory [20]. The table below reports study participant demographic data.

### 2.2 Healthy Controls

The control group consisted of 9 healthy patients (4 M, 5 F median age of 42.22 ± 2 years), with no pain, no current or previous history of a relevant neurological or psychiatric disease, and no current regimen of any medications known to affect the EEG signal.

### 2.3 Experimental Protocol and Data Collection

The study was approved by the University of Oklahoma Institutional Review Board, IRB # 14252. All patients were informed regarding study aims, scope, and were provided written informed consent. Patients were seated comfortably in a distraction free room and told to maintain alert wakefulness, avoid unnecessary movements including talking over a period of 3 minutes with eyes open. Between recording sessions, EEG equipment was calibrated and on a per channel basis, and impedances were maintained below 50 kΩ. On the day of recording, patients were advised to abstain caffeine to avoid induced EEG theta frequency band power changes [21]. EEG signals were measured with 64 Ag/AgCl surface electrodes, fixed within a standard EEG cap according to the 10–20 EEG system [22]. EEG signals were registered using the Brainvision EEG system (Neuroscan Compumedics, Houston, TX, 16-bit A/D conversion, at a sampling frequency of 5000 Hz, 0.5 Hz–100 Hz band pass filter, and 0.2 seconds time constant) and data was continuously viewed on a PC monitor.

### 2.4 EEG Pre-Processing

Once collected, EEG data was processed in MATLAB R2023a (MathWorks, Natick, MA, USA) with custom EEGLAB toolbox scripts to convert data from raw EEG files to MATLAB compatible arrays [23]. Subsequently, the matrices were further analyzed using custom made MNE Python 3.10.9 scripts [24]. A neutral virtual reference was computed to standardize the reference of the EEG recordings via the Reference Electrode Standardization Technique [25]. EEG recordings were band-pass filtered from 1 to 45 Hz. Using the Parks-McClellan algorithm, the optimal Chebyshev finite impulse response filters were designed with customized order for error minimization pass and stop bands to remove signal drift and 60 Hz noise [26]. Physiological noise including heartbeat and respiration was removed from the EEG signal with a cutoff frequency of 0.2 Hz. Ocular artifacts including eye movement and blinking were removed from the EEG time series using independent component analysis (i.e., FastICA algorithm) [27]. Visual inspection of the results was based on the topography and time course of the component, and retained component EEG data was re-referenced. Spatial ICA components extracted from 1–45 Hz EEG data were applied to 1–500 Hz EEG data via the unmixing ICA matrix; components were rendered, visually inspected, and removed. Welch’s power spectral density was computed for each EEG channel using time window duration of 8 seconds and corresponding frequency resolution of 0.125 Hz. Corresponding logarithmic coordinate plots (i.e., log-power vs log-frequency) were used to estimate brain activity. Using the mean of the power spectral densities obtained for all channels, the global power spectral density was subsequently calculated. Band powers were computed in the following physiological EEG frequency bands: delta, theta, alpha, beta, gamma. EEG frequency envelopes were extracted from the above data within the following ranges [delta: 0.5 – 4 Hz, theta: 4 – 8 Hz, alpha: 8 – 13 Hz, beta: 14 – 30 Hz, and gamma: 30 – 100 Hz] using a FIR bandpass filter.

Using the equation below, we estimated spectral entropy (SE) as:

(1) $$SE=-\sum_{f_{min}}^{f_{max}}rPS\left(f\right){log}_{2}rPS(f)$$

where $f_{min}=\;0.5\;\mathrm{Hz}$ and $f_{max}=\;100\;\mathrm{Hz}$. SE provides an index of the amount of relative power spectrum (i.e., power spectrum fragmented in frequency components) with respect to total power, thereby quantifying the robustness of the spectrum. Specifically, in the EEG time series while considering all frequencies, white noise power spectrum is constant with maximal entropy and all frequencies have the same weight.

### 2.5 Hurst Fractal Dimension Measure

Recently, nonlinear measures have been developed to further the understanding of the human brain’s inherent chaos [28]. One such measure is the Hurst exponent, which can be interpreted as a central tendency estimator of a time series [16], [29], [30]. Typically, local variation with respect to global oscillation is viewed through the lens of the Fractal Dimension (FD). Other well-known estimators that evaluate the sensitivity of initial conditions time series (i.e., Lyapunov exponent), typically do not have linear equivalence, a characteristic unique to chaotic systems [31]. As the brain is a complex dynamical system, it remains in a permanent state of oscillation between organized and chaotic functional structures [29], [32].

For a given time series, the Hurst Fractal Dimension (HFD) exponent evaluates the degree of self-similarity, based on the comparison of oscillatory structure of the complete series with itself divided into successive parts. This formalism leads to a rescaled analysis allowing for the approximation of the slope of a log-log time interval plot with values varying between zero and unity [16]. Assuming a threshold Hurst exponent value of 0.5, series with Hurst exponents falling below this threshold suggests that the time series tends toward stability while continuing a state of chaotic steady oscillation around a relatively narrow range of values with time [15] and are categorized as an anti-persistent or short-memory series with oscillation around a central attractor value over time with a homeostatic complex memory [16], [30]. Hurst values greater than 0.5 are assumed to follow the Hurst Effect [15]–[17], [30], [33]. The Hurst Effect describes movement away from a centralized stabilizing value, thereby repeating patterns that precede said value, move away from, or approach chaos. When values are close to H=0.5, they represent the midpoint between chaos (i.e., random walk or Brownian motion type oscillations; H values closer to zero), and order (H values closer to unity) [15], [29], [34]. In our experiments, the HFD exponent was computed for each of the EEG segments and for each EEG channel in 30 second time windows (i.e., 0, 30, 60, 90, 120, 150) as proposed by Hurst [15], [17], [30], [33], [35]. From each participant, the resulting EEG resting state non-overlapping segments consisted of $5.7\;x\;10^{7}$ data points (i.e., 63 channels, 5000 Hz, 180 seconds) for a grand total of 138 EEG segments (i.e., from chronic pain and healthy control patients).

### 2.6 Statistical Analysis

We implemented statistical analyses to determine statistical differences of HFD values between chronic pain and healthy control patients in all EEG channels. Data was categorized in the following groups: full frequency EEG signals and EEG frequency bands. Subgroups from each group represent healthy control patients, and chronic pain patients. Fractal dimension values associated with EEG frequency bands and corresponding spectral characteristics were correlated between groups. The Wilcoxon Signed-Rank test (p<0.05), the Kruskal–Wallis test, and repeated measure n-way ANOVA testing were implemented to determine inter and intra-group HFD statistical differences. Pearson’s correlation for HFD and EEG band power was computed and when appropriate, Bonferroni correction for multiple comparisons was applied, see Figure 8 in the Appendix. Due to the power distribution of the EEG alpha band, we ignore analysis in the 8–13 Hz range. To further test the reliability of our results, we randomized the EEG time series via k-fold shuffling (k=10) and computed the Hurst fractal measure and tests for statistical significance as described above [36]. Specifically, all data was randomly divided into ten equal size groups. One group was retained for validation testing and the remaining nine groups were used for determining the HFD. This procedure was repeated ten times and the testing group was used only once. The HFD was evaluated by averaging the results from the ten testing groups.

## 3. Results

### 3.1. HFD in the Frontal Cortex

**Figure 1.**
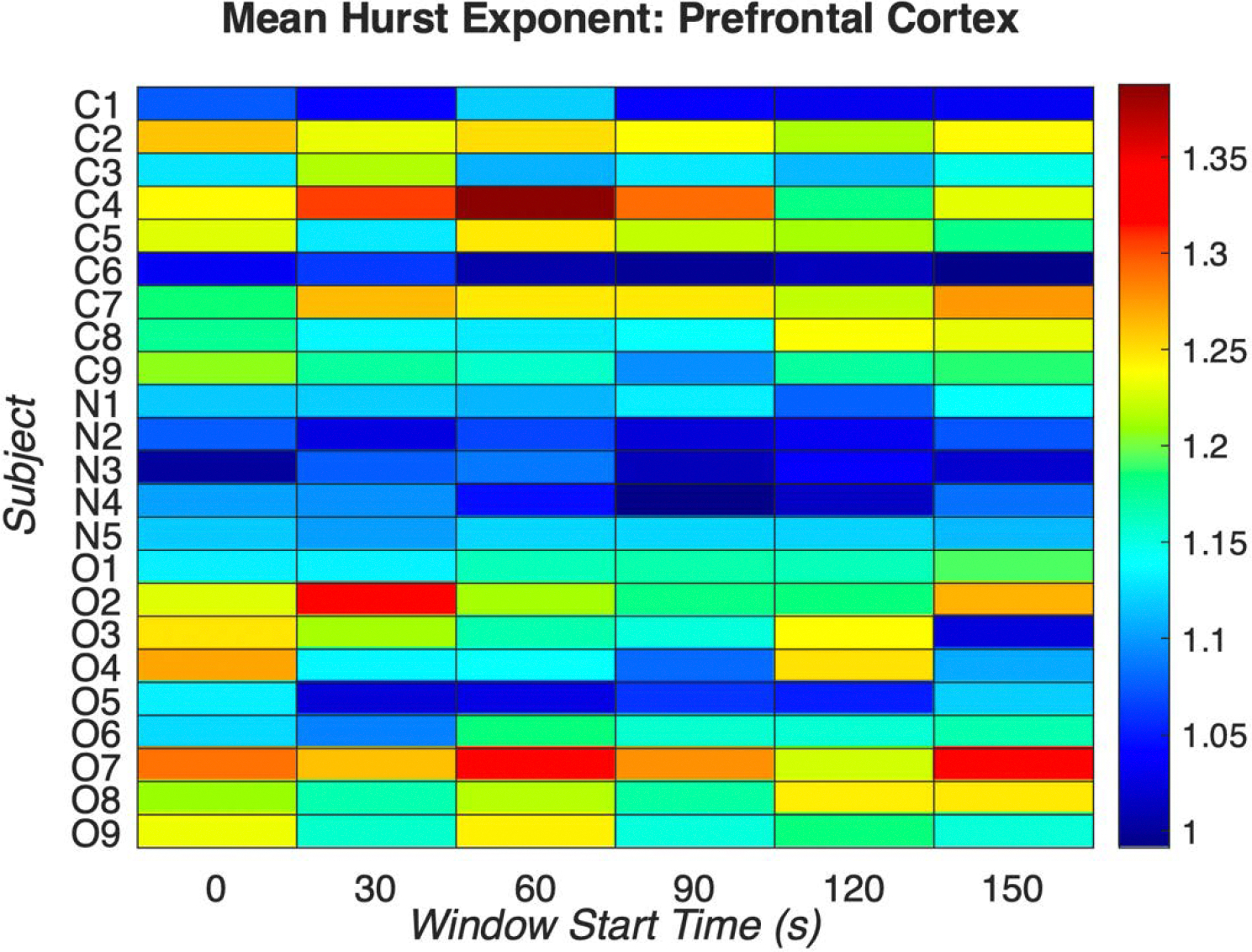
Mean Hurst exponent indices for prefrontal EEG electrodes across all groups using full spectrum EEG signals. “C”, “N”, and “O” refer to control patients, patients who have chronic pain but not actively taking opioid medications, and patients with chronic pain and are actively taking opioid medications respectively. For each window start time, note the patients with chronic pain but not taking opioid medications (N1-N5) have similar Hurst exponent values compared to the other two groups. In the “C”, control group, the mean Hurst exponent is 1.1671, range: 0.9913–1.3875, standard deviation 0.0902. In the “N”, non-opioid group, the mean Hurst exponent is 1.0745, range: 0.9922–1.1405, standard deviation 0.0446, and in the “O”, opioid group, the mean Hurst exponent is 1.1804, range: 1.022–1.338, standard deviation 0.0748. The mean Hurst exponent value corresponding to the “N”, non-opioid group suggests lower chaos as compared to other groups. There is statistical significance (p<0.05) between the “C” and “O” groups suggesting that the opioid group has dissimilar chaos as compared to the control group.

Using multifractal analysis and particularly the GHE method, we reveal nonlinear and complex dynamics in resting state EEG recordings of chronic pain patients actively taking opioid medications and those not taking opioid medications. Results from this analysis help to better understand phenomenology and enhanced distinctions between signals from healthy, chronic pain, and opioid dependent patients. It is well known that the prefrontal cortex of the human brain plays a significant role in pain processing and addiction [37]. Activity from left, right and sagittal (midline) areas of the prefrontal cortex were analyzed to examine brain wide scale free fractal activity from full spectrum EEG. We computed the mean Hurst exponent for each window (0, 30, 60, 90, 120, 150). We note clear evidence of multifractality in both EEG signals of healthy patients and opioid dependent patients across sensors and similar patterns of fractal activity persist across groups. The mean Hurst exponent values are shown in Figure 1 from all prefrontal cortex EEG channels as an illustrative example of pain processing and fractal spatial sensitivity. The values are calculated from all subjects representing all groups, i.e., the control group, the opioid group, and the non-opioid group. The mean Hurst exponent across the control and opioid dependent groups shows heterogeneity as compared to the non-opioid group. This suggests that the non-opioid group is less persistent compared to both the control and opioid groups and has same characteristic value independent of the scale at which the time series is examined. Hurst exponent values corresponding to the “N” group in Figure 1 show this feature, suggesting persistence as the data structure preserves statistical integrity even if modified.

### 3.2 Group Parameters and HFD

**Figure 2.**
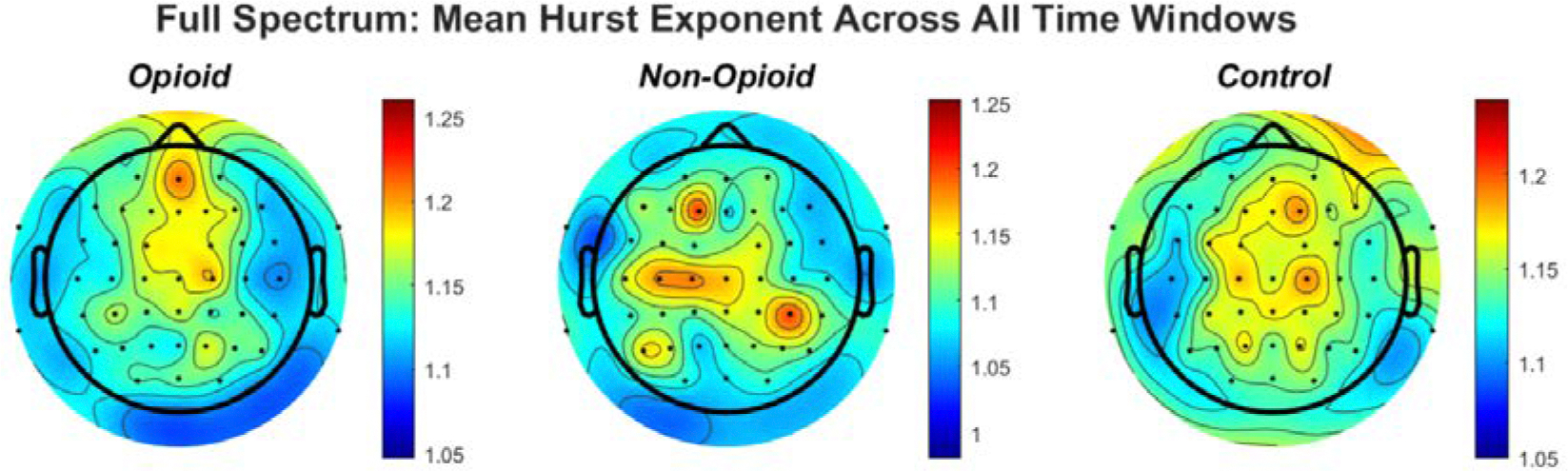
The topographic plots of mean values of the Hurst exponent across groups are shown. The circles within the plots represent the EEG electrodes. In the opioid and control groups, similar patterns emerge for HFE values across the brain. HFE values increase in EEG electrodes representing the frontal and parietal lobes of the brain and decrease in the temporal and occipital lobes. The control topographic plot shows higher HFE values in the prefrontal cortex as compared to other brain regions.

Multifractal analysis and determination of the Hurst fractal dimension revealed topographic specificity across and within groups of EEG signals. Statistical analysis via the Kruskal–Wallis test, Wilcoxon sign ranked test, n-way ANOVA and paired t-test suggested statistical significance across groups, sex, handedness, and EEG frequency bands. A strong correlation was found between full EEG frequency signals and HFD in the chronic opioid groups as compared to healthy controls. Similar comparisons between the computed Hurst exponent values and all EEG frequency bands are shown in the Appendix. Figure 2 shows the topography of the Hurst fractal exponent across all groups and full frequency EEG signals. Statistical analysis of group population parameters including sex and handedness with respect to full spectrum and specific EEG frequency bands was computed. Statistical results are shown in table 2 below. Figure 8 in the Appendix shows the confidence intervals from our n-way ANOVA (n = 5) statistical analysis of the HFD for the combinations of groups, handedness, EEG frequency content, sex, and sensor location, we note that there is a strong relationship between HFE values and group parameters derived from opioid and control groups in full spectrum and higher EEG frequency signals. Table A1 in the appendix shows N-way ANOVA interactions and statistical significance values between all combinations.

## 4. Discussion

Clinically, the EEG remains an essential tool for the diagnosis of neurological disorders and mathematical approaches utilizing multifractal analysis can help characterize complexity in brain disorders. In this work, the complexity of EEG recordings collected from 23 patients consisting of two groups; chronic pain and healthy controls (and subgroups: one taking opioid medications and one not taking opioid medications) were evaluated by means of multifractal analysis, and computation of the Hurst exponent. An explicit purpose of our study was to determine a non-invasive reliable fingerprint to distinguish opioid use from healthy control EEG signals. The derived Hurst exponent values provide motivation on the underlying memory present in such signals, providing effective discrimination between EEG signals belonging to healthy control subjects and opioid dependent chronic pain patients.

Using parametric and non-parametric statistical tests, we test if the obtained robust estimates of the generalized Hurst exponent between groups are statistically significantly different. Our battery of statistical tests shows evidence of statistical significance between generalized Hurst exponent estimates obtained from EEG records of control and chronic pain patients, as shown in Table 2. Furthermore, the estimated Hurst exponents from healthy patients are of lower magnitude as compared to the chronic pain groups with or without the use of opioid medications, across time windows. Therefore, short term and longer-term dynamics in healthy EEG signals show similar persistence to EEG signals in opioid using patients. Our findings suggest that multifractal analysis particularly the computation of GHE aids in understanding short and long variations in EEG signals as neural activity engages nonlinear dynamic mechanisms of unique synchronous brain electrical impulses in the opioid dependence. The GHE provides information regarding improved assessment of long-term autocorrelation (i.e., memory) in EEG signals associated with opioid dependence. Higher EEG frequency bands show GHE-based multifractal estimates to be more robust and appropriate signal patterns that can be used to characterize dynamics in healthy controls and in opioid dependent groups as compared to other EEG frequency bands.

When comparing Hurst values for all EEG frequencies, values greater than the established threshold, T (i.e., 0.5), correspond to gamma band frequencies between 30–90 Hz in the ‘N’ and ‘O’ groups. Hurst values of the beta EEG frequency band predominate with Hurst values greater than those corresponding to the healthy control group, suggesting the impact of the Hurst effect globally to execute cyclic, regular, predictable, and persistent functions in the short and mid-term of the time series [1], [11], [13]. Figure 1 shows the mean Hurst exponent indices for prefrontal EEG electrodes across all groups using full spectrum EEG signals. In basal resting conditions, the H-values of the beta and gamma bands predominate over the others. In the relative short medium term, H > 0.5 confers to the whole brain the Hurst effect necessary to perform cyclic, regular, and persistent functions, accepting a certain degree of predictability. For other bands, lower Hurst values indicate anti-persistent processes in early windows and suggest persistent stability in later windows. This accounts for resorting to a central tendency value, suggesting fast information storage and processing in response to opioid dependence [38], [39].

**Figure A1.**
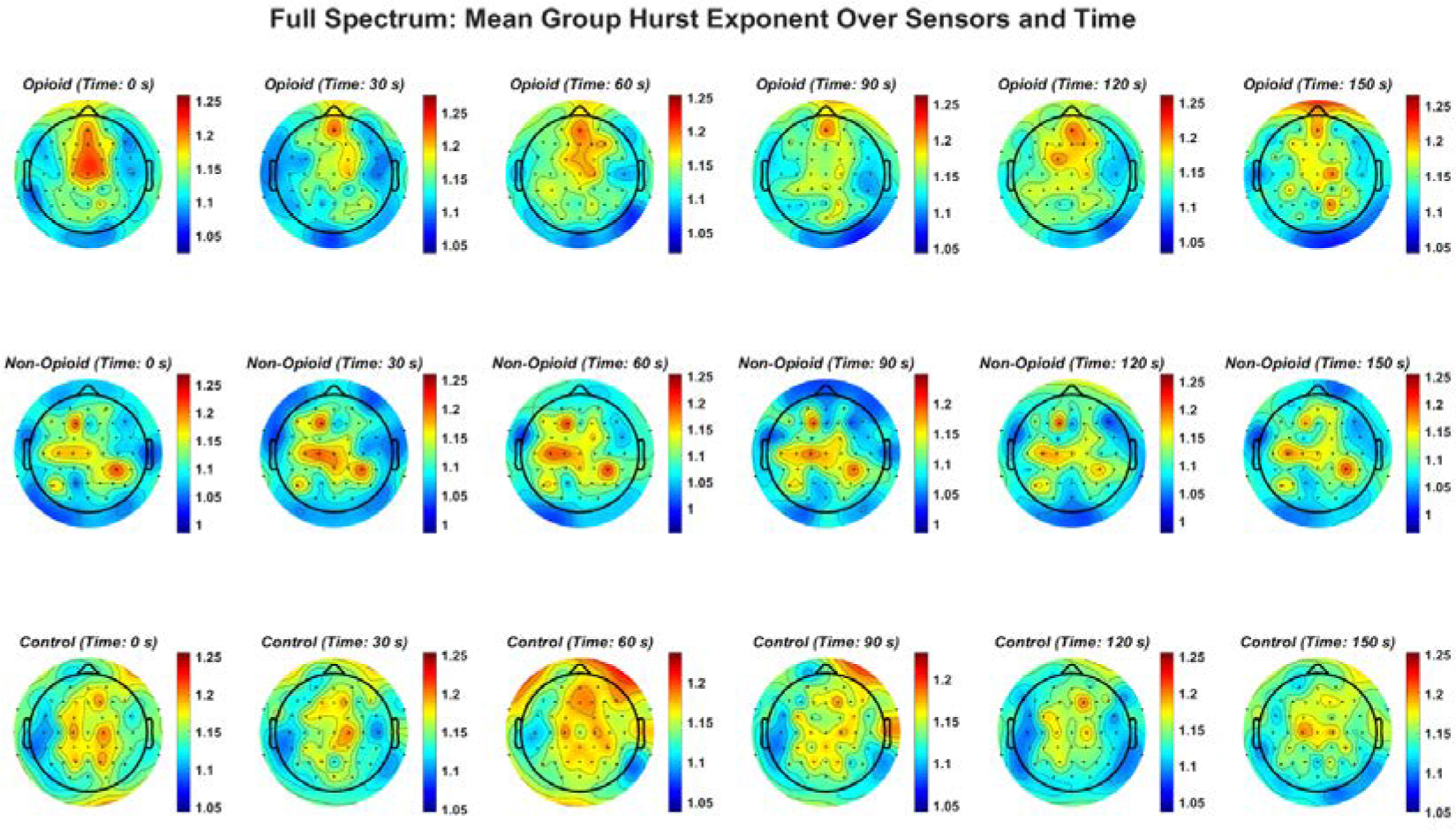
The mean Hurst exponent for all chronic pain patients taking opioids, chronic pain patients not taking opioids, and healthy control patients across all time windows and EEG sensors using full frequency spectrum EEG data is shown. Note that HFD values are relatively lower for full frequency signals in all groups as compared to all other bands (1.0 to 1.25).

**Figure A2.**
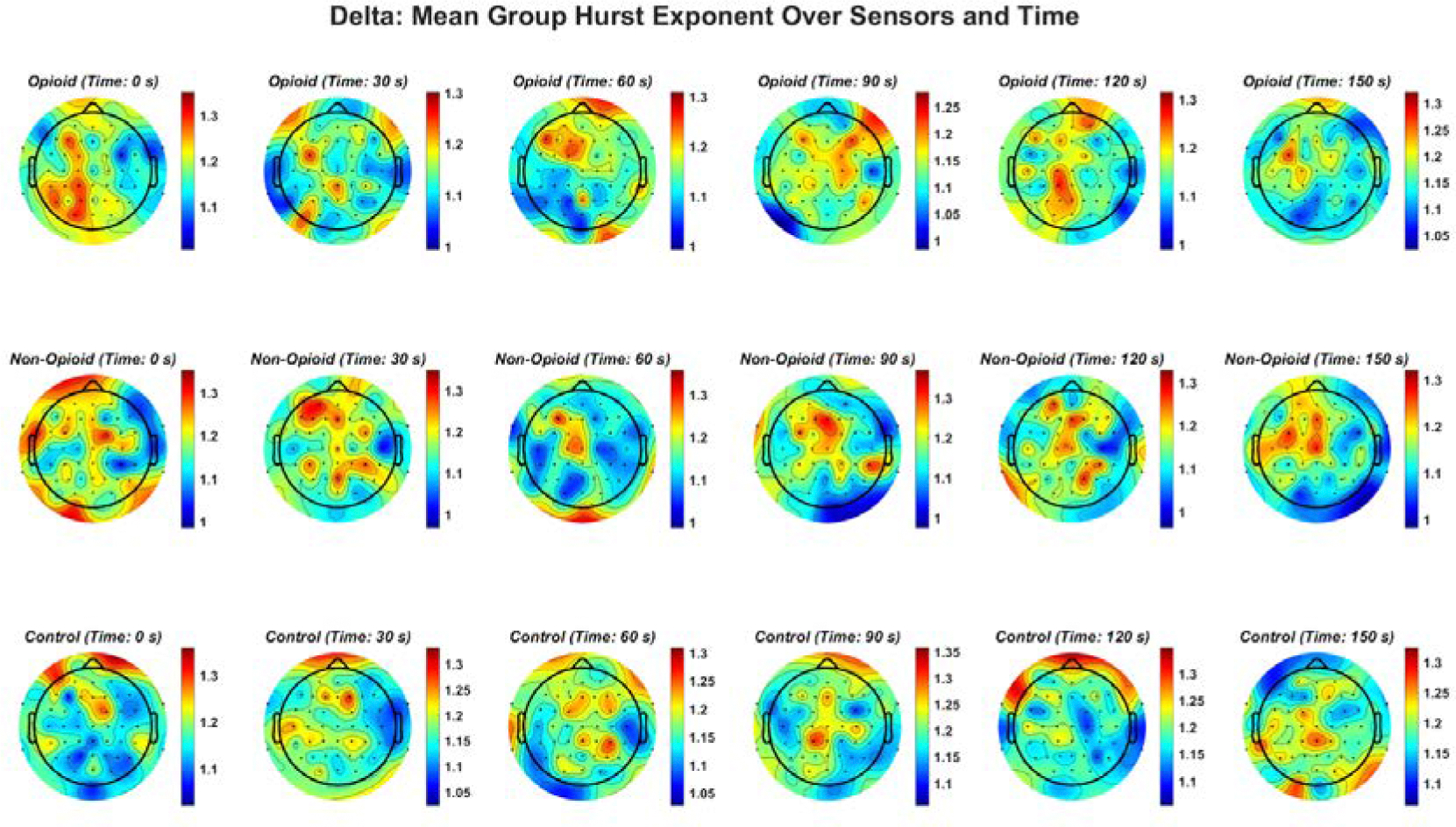
EEG delta frequency band and the Hurst exponent value across all groups. Fractal distribution pattern is similar across control and opioid groups in the frontal and temporal areas.

**Figure A3.**
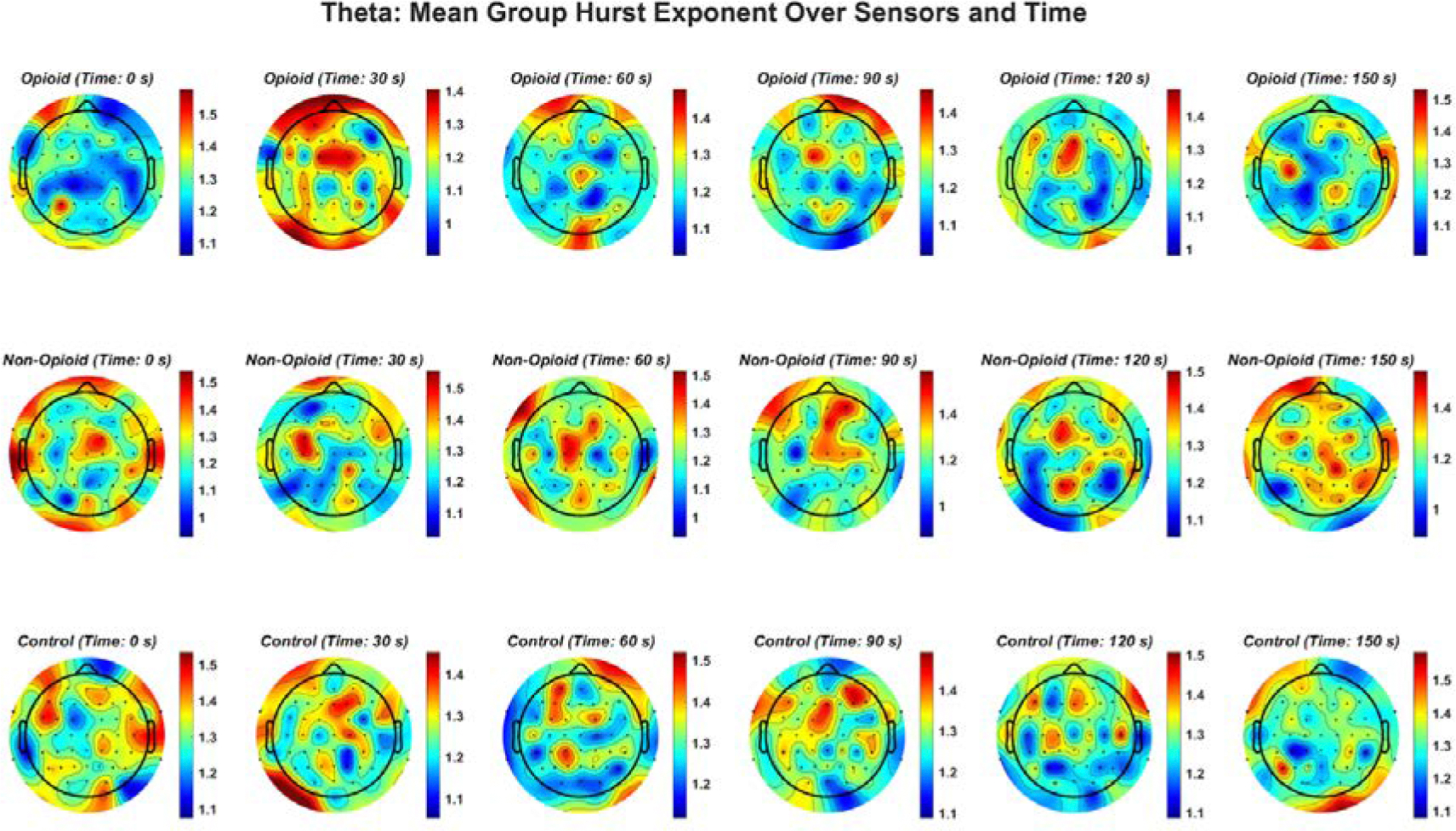
EEG theta frequency band and the Hurst exponent value across all groups. Fractal distribution pattern is similar across control and opioid groups in the frontal and temporal areas.

**Figure A4.**
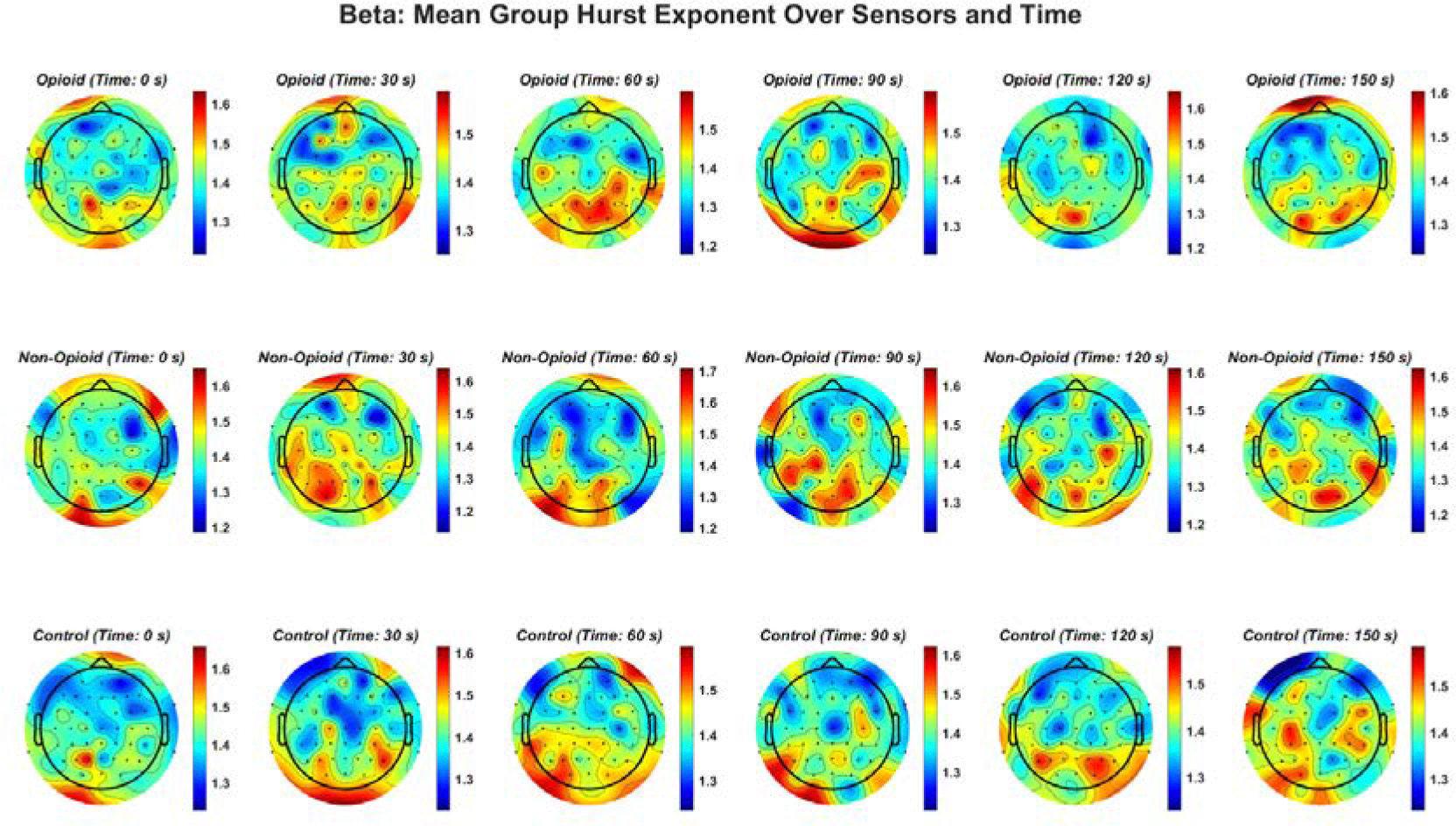
EEG beta frequency band and the Hurst exponent value across all groups. Higher EEG frequency bands (beta, gamma), demonstrate a similar pattern in non-opioid group and controls.

**Figure A5.**
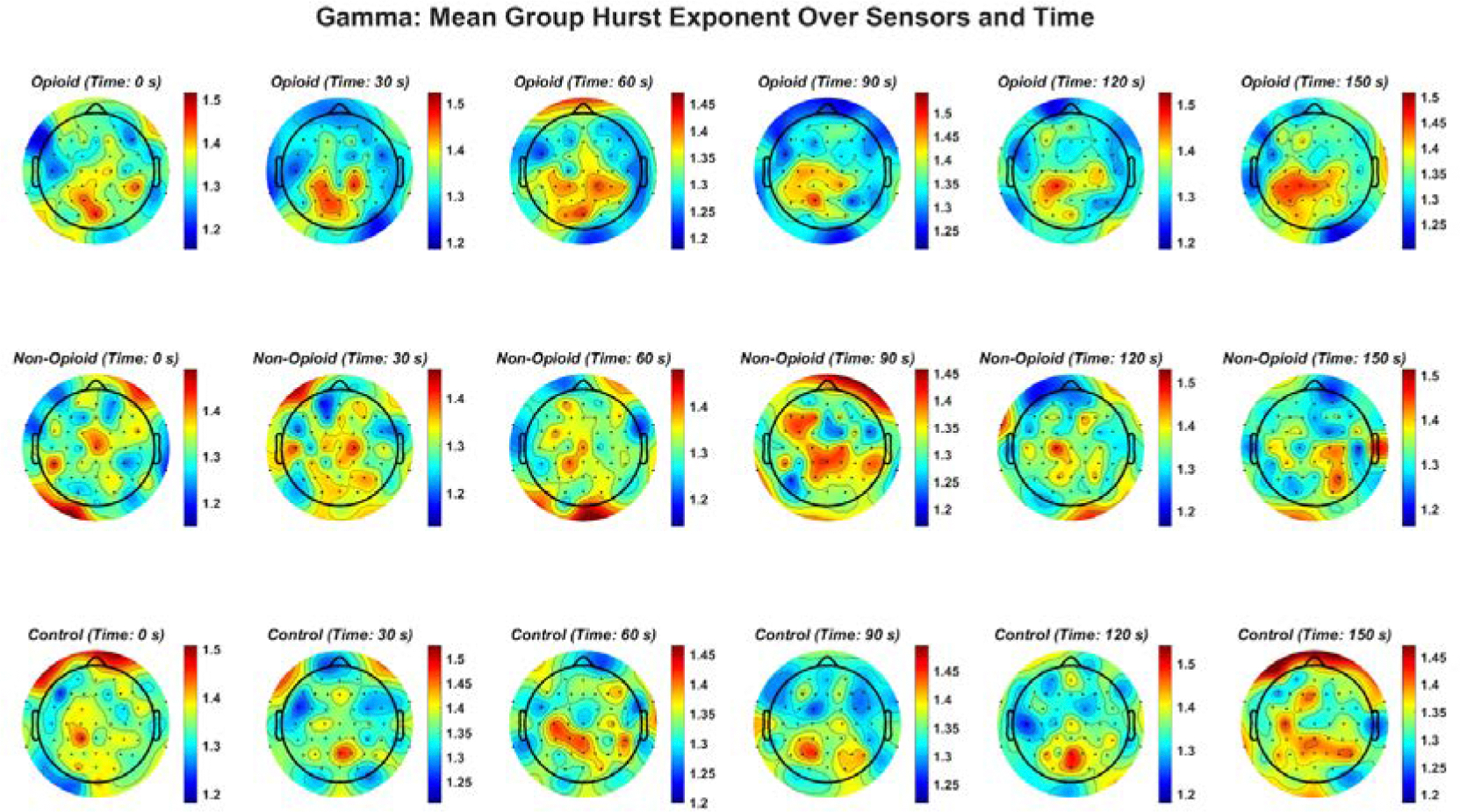
EEG gamma frequency band and the Hurst exponent value across all groups. EEG beta frequency band and the Hurst exponent value across all groups. The gamma frequency band demonstrates a similar pattern in non-opioid group and controls.

**Figure A6.**
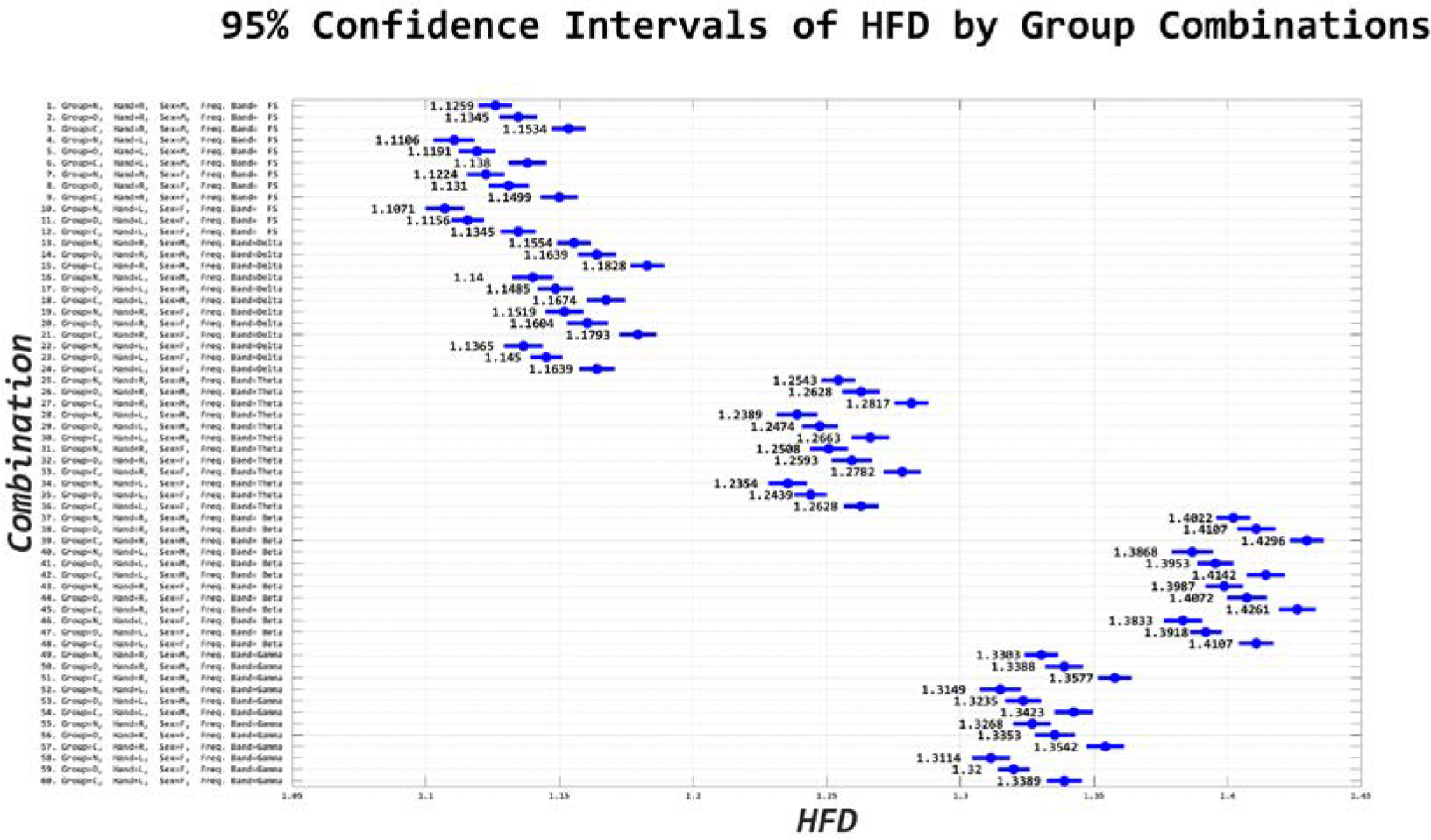
Confidence intervals derived from n-way ANOVA statistical testing with multiple comparison correction using the Bonferroni method. The blue circles and corresponding number value represent the mean and range of the HFD. There are 60 combinations consisting of full spectrum EEG and individual EEG frequency bands with group, sex, and handedness, as derived from the Edinburgh Handedness Inventory are shown. ‘FS’ refers to full spectrum EEG signals. Note that lower EEG frequency bands are anti-persistent and higher EEG frequency bands (i.e., beta and gamma bands) correspond to a higher HFD value suggesting that they have higher tendencies to regress to the mean (i.e., anti-persistent).

We analyzed localization of HFE values across the brain spatially in all groups. Figure 2 shows the topographic representation of mean values of the Hurst exponent across all groups. We note that in the opioid and control groups, similar patterns emerge for HFE values across the brain. HFE values increase in EEG electrodes representing the frontal and parietal lobes of the brain and decrease in the temporal and occipital lobes. The control topographic plot shows higher HFE values in the prefrontal cortex as compared to other brain regions, confirming localization of addictive potential in the brain [8], [12], [37]. It can be postulated that chronic pain and opioid use provides an impetus for neural circuit reorganization and in these situations, the presence of scale free patterns changes with brain reorganization [17], [33], [40] [41]. Finally, we computed HFE values for each EEG frequency band with respect to group population parameters (sex and handedness). Figure A6 shows the distribution of confidence intervals of our statistical analysis of the combinations between groups, handedness, EEG frequency content, sex, and EEG sensor location. Previous neuroimaging pain studies have determined the existence of a distributed pain matrix across hemispheres with typical cortical pain processing lateralizing toward the right hemisphere [42]. Our results show that subject handedness impacts pain processing as there is a significant difference in HFE values for patients who are righthanded versus lefthanded consistent across sensor location, sex, and frequency band (i.e., combinations: [non-opioid group, right-handed, male] and [non-opioid group, left-handed, male]) in Figure A6. Furthermore, preferential hemispheric activation leads to bilateral or contralateral activation of the pain matrix. Spatial localization of brain regions remains consistent with the prevailing paradigm that pain stimuli is alerted by a preferred lateralization attention system [42]. Our results in Figures A2 suggest lower HFD values correspond to low frequency oscillations (i.e., delta EEG frequency band) possibly representing trivial unchanging oscillations over time and higher EEG frequency bands (i.e., gamma) correspond to higher HFD values (Figure A3, A4, A5). In addition, the HFD index of localized brain activity corresponding to the frontal lobe (i.e., pain processing center) is of higher magnitude as compared to other brain areas (i.e., temporal, and occipital brain lobes) when examining the full EEG frequency spectrum (Figure A1). By examining the self-similarity of the EEG signal in distinct frequency bands with diverse amplitude-time characteristics, the fractal dimension aids in quantifying the correlation between frequency range and brain activity in pain. The multifractal analysis approach aids in quantifying the characteristics of the EEG in opioid use. The analogous processes corresponding to non-opioid use can be determined, whereby this similarity can be explained by scale invariance. Results here show that self-similar behavior in full spectrum, low and high EEG frequency bands allow for the determination of the dimensionless ratio characterized by its fractal dimension (i.e., Hurst dimension). The proposed methodology here can be leveraged within other mathematical, or machine learning frame works in future work as scale free fractal properties are expected to help characterize the high dimensional nature of neural dynamics associated with efficient brain signal processing.

## Figures and Tables

**Table 1.** Study participant demographic data. “C”, “N”, and “O” refer to control patients, patients who have chronic pain but not actively taking opioid medications, and patients with chronic pain and are actively taking opioid medications respectively. “R” and “L” refer to right and left handedness subjectively.

Participant	Age	Sex	Opioid Status	Handedness
C1	59	F	N	R
C2	61	F	N	R
C3	60	F	N	R
C4	52	F	N	R
C5	36	M	N	R
C6	25	M	N	R
C7	29	F	N	L
C8	28	M	N	R
C9	32	F	N	R
N1	73	M	N	R
N2	66	F	N	R
N3	55	F	N	R
N4	92	F	N	R
N5	39	M	N	R
O1	58	F	Y	R
O2	75	M	Y	R
O3	47	F	Y	R
O4	39	F	Y	R
O5	70	F	Y	L
O6	74	F	Y	L
O7	64	F	Y	L
O8	47	M	Y	R
O9	47	F	Y	R

**Table 2.** Statistical analyses of groups, group parameters, and EEG frequency bands. Results show statistical significance for control, non-opioid, and opioid groups as well as tested group parameters and EEG frequency bands.

Statistical analysis	Parameter				
	Group	Handedness	Sex	Sensor localization	EEG frequency band
n-way ANOVA	*p-value*	*p-value*	*p-value*	*p-value*	*p-value*
	8.8130e-46	1.2406e-11	0.0479	5.9527e-10	0
	*F-statistic*	*F-statistic*	*F-statistic*	*F-statistic*	*F-statistic*
	103.9907	45.9308	3.913	21.2524	4.4963e4
Paired t-test	*p-value*	*p-value*	*p-value*	*p-value*	*p-value*
	N/A	4.2005e-13	1.7936e-4	N/A	N/A
	*t-statistic*	*t-statistic*	*t-statistic*	*t-statistic*	*t-statistic*
	N/A	7.2512	3.7468	N/A	N/A
Kruskal-Wallis	*p-value*	*p-value*	*p-value*	*p-value*	*p-value*
	7.6782e-37	1.5328e-15	7.0521e-5	7.0059e-8	0
	*Chi-square*	*Chi-square*	*Chi-square*	*Chi-square*	*Chi-square*
	166.3145	63.5891	15.7968	32.9479	1.5035e4
Wilcoxon Signed Rank Test	*p-value*	*p-value*	*p-value*	*p-value*	*p-value*
	N/A	1.5328e-15	7.0521e-5	N/A	N/A
	*z-statistic*	*z-statistic*	*z-statistic*	*z-statistic*	*z-statistic*
	N/A	7.9743	3.9745	N/A	N/A

## Data Availability

The data presented in this study are available on request from the corresponding author.

## Appendix A

The mean of the computed HFD is shown in the following figures for full EEG frequencies and specific EEG frequencies. In all figures we note the behavior of the mean HFD is heterogenous across EEG frequency bands, implying a multifractal nature in each band. For each time window, there are clear differences between HFD. We observe that HFD values are relatively lower for full frequency signals in all groups as compared to all other bands (1.0 to 1.25), as shown in Figure 3 below. In the lower bands (delta, theta) similar distribution of the HFD is seen in control and opioid groups in the frontal and temporal areas, whereas in higher bands (beta, gamma) a similar pattern is seen in non-opioid group and controls after the 90 second time window (Figures 4,5,6,7).

Short term dynamics in healthy control full frequency and enveloped EEG signals have increased persistence compared to opioid dependent and non-opioid dependent chronic pain groups. For time windows below 90 seconds, the full spectrum EEG signals from both chronic pain groups (opioid and non-opioid dependent) exhibit identical dynamics. For instance, at higher time windows (120, 150 seconds) the distribution of the Hurst exponent changes across all groups, while remaining in the same range. In other words, long term dynamics in EEG signals across the control and non-opioid dependent brain are temporally and spatially persistent, while long term dynamics in opioid EEG signals are temporally and spatially anti-persistent [4], [14], [43].

The mean HFD is spatially distributed and is different among healthy and chronic pain patients is further confirmed by visual inspection as well as from our battery of statistical tests: Kruskal–Wallis test, Wilcoxon sign ranked test, paired t-test, and n-way ANOVA. The null hypothesis associated with each statistical test is rejected (pvalue<0.05), and we conclude that healthy EEG signals have a unique fractal as compared to chronic pain opioid free and opioid dependent groups.

**Table A1.** N-way ANOVA interactions between all combinations are shown in the table. Group refers to C, N, O, handedness refers to left and right handedness, Sex refers to male, female, sensor location refers to the left, right, and midline regions of the brain, and frequency band refers to full spectrum and delta, theta, beta, and gamma frequency band.

Source	d.f.	F-value	p-value
Group	2	103.99072	8.81304E-46
Handedness	1	45.930828	1.24062E-11
Sex	1	3.9130327	0.047919367
Sensor Location	2	21.252392	5.9527E-10
Frequency Band	4	4496.2684	0
Group: Handedness	2	9.0744042	0.000114778
Group: Sex	2	48.19405	1.23817E-21
Group: Sensor Location	4	11.963476	1.02468E-09
Group: Frequency Band	8	133.03556	1.1238E-221
Handedness: Sex	1	116.09799	4.8946E-27
Handedness: Sensor Location	2	11.930848	6.60574E-06
Handedness: Frequency Band	4	129.95736	1.5922E-110
Sex: Sensor location	2	9.4308948	8.03718E-05
Sex: Frequency Band	4	109.93517	2.1452E-93
Sensor Location: Frequency Band	8	166.06096	3.1492E-277
Group: Handedness: Sex	2	119.86895	1.21567E-52
Group: Handedness: Sensor Location	4	25.04814	9.4611E-21
Group: Handedness: Frequency Band	8	105.34079	6.7935E-175
Group: Sex: Sensor Location	4	3.2394849	0.011491284
Group: Sex: Frequency Band	8	114.83201	6.0527E-191
Group: Sensor Location: Frequency Band	16	93.512548	7.8574E-304
Handedness: Sex: Sensor Location	2	94.353797	1.29266E-41
Handedness: Sex: Frequency Band	4	74.442126	5.41974E-63
Handedness: Sensor Location: Frequency Band	8	5.8322719	1.79865E-07
Sex: Sensor Location: Frequency Band	8	47.625371	4.83496E-77
Group: Handedness: Sex: Sensor Location	4	22.371579	1.76906E-18
Group: Handedness: Sex: Frequency Band	8	139.54489	1.2061E-232
Group: Handedness: Sensor Location: Frequency Band	16	92.127707	3.1792E-299
Group: Sex: Sensor Location: Frequency Band	16	92.937352	6.4236E-302
Handedness: Sex: Sensor Location: Frequency Band	8	356.1026	0
Group: Handedness: Sex: Sensor Location: Frequency Band	16	143.17433	0
